# Berberine promotes host lipolysis to enhance antimicrobial defense against hypervirulent *Klebsiella pneumoniae* infection

**DOI:** 10.71150/jm.2605001

**Published:** 2026-08-31

**Authors:** Ju Yeong Lee, Hui-Jung Jung, Anwesha Ash, Seunghyeon Jeon, Miri Hyun, Ji Yeon Lee, Sang-Hee Lee, Hyuk Nam Kwon, Won-Ki Baek, Jichan Jang, Hyun ah Kim, Jin Kyung Kim

**Affiliations:** 1 Department of Microbiology, Keimyung University School of Medicine, Daegu 42601, Republic of Korea; 2 Division of Life Science, Department of Bio & Medical Big Data (BK21 Four Program), Research Institute of Life Science, Gyeongsang National University, Jinju 52828, Republic of Korea; 3 Department of Infectious Diseases, Keimyung University Dongsan Hospital, Keimyung University School of Medicine, Daegu 42601, Republic of Korea; 4 Center for Bio-imaging & Translational Research, Korea Basic Science Institute, Cheongju 28119, Republic of Korea; 5 Department of Chemistry, Hanyang University, Seoul 04763, Republic of Korea; 6 Department of Biological Sciences, University of Ulsan, Ulsan 44610, Republic of Korea; 7 Basic-Clinic Convergence Research Institute, University of Ulsan, Ulsan 44610, Republic of Korea

**Keywords:** hypervirulent *Klebsiella pneumoniae*, berberine, lipid droplet, lipolysis, TFEB

## Abstract

Hypervirulent *Klebsiella pneumoniae* (hvKp) is an emerging pathogen that causes severe community-acquired infections; however, the immune mechanisms controlling intracellular hvKp have yet to be clearly defined. In this study, we investigated the therapeutic potential of berberine against hvKp infection and elucidated the underlying molecular mechanisms using macrophage cell models and in vivo zebrafish models. Berberine significantly reduced intracellular hvKp survival in macrophages and improved survival in hvKp-infected zebrafish. Berberine markedly attenuated proinflammatory cytokine production and inhibited the c-Jun N-terminal kinase (JNK) and extracellular signal-regulated kinase (ERK) signaling pathways. Notably, we found that hvKp exploited host lipid droplets (LD) biosynthesis to support its intracellular survival, and berberine effectively suppressed LD accumulation. Mechanistically, berberine promoted the nuclear translocation of transcription factor EB (TFEB), thereby enhancing lipolysis. Although berberine upregulated autophagy-related gene expression during hvKp infection, it did not induce lipophagy, the selective autophagic degradation of LD. Collectively, these findings indicate that berberine has potential as a therapeutic agent against hvKp infection by modulating host lipid metabolism to restrict bacterial intracellular survival.

## Introduction

*Klebsiella pneumoniae* (Kp) is an opportunistic Gram-negative bacterium belonging to the *Enterobacteriaceae* family that primarily colonizes the human gastrointestinal tract and nasopharynx. It is also widely found on the mucosal surfaces of animals and in natural environments, such as water and soil (Wang et al., 2020). In immunocompromised individuals, it can invade the host and cause a wide range of severe infections, including pneumonia, urinary tract infections, and sepsis (Abbas et al., 2024). The increase in multidrug-resistant Kp strains has become a critical public health concern, leading to high morbidity and mortality worldwide (Mohd Asri et al., 2021). Among these strains, hypervirulent Kp (hvKp) has emerged as a clinically significant pathogen with higher virulence than classical Kp (cKp) (Russo and Marr, 2019). HvKp is characterized by a hypermucoviscous polysaccharide capsule that helps the bacterium to evade host immune defenses (Liu et al., 2025; Xu et al., 2021) and allows it to cause invasive infections, even in healthy individuals. Furthermore, hvKp strains that acquire antibiotic resistance pose a serious threat to public health because of their enhanced ability to spread in both community and healthcare settings (Marr and Russo, 2019). Considering the clinical importance of hvKp, a comprehensive understanding of host defense mechanisms against this pathogen is vital for developing effective therapeutic strategies.

Berberine is a plant-derived isoquinoline alkaloid with a long history of use in traditional medicines (Neag et al., 2018). Recent studies have highlighted its broad pharmacological effects, including anti-inflammatory, antidiabetic, and lipid-regulatory properties (Cai et al., 2023; Neag et al., 2018). In macrophages, berberine has been shown to modulate lipid metabolism, which plays a critical role in the maintenance of homeostasis and host defense (Li et al., 2017). This modulation is particularly relevant in the context of hvKp infections, in which macrophage function is compromised. Therefore, understanding how berberine influences lipid metabolism in macrophages during hvKp infection may provide insights into potential therapeutic strategies.

Lipid droplets (LD) are intracellular organelles that store neutral lipids, such as triacylglycerols (TG) and cholesterol esters, surrounded by a phospholipid monolayer embedded with LD-associated proteins (Olzmann and Carvalho, 2019). Proper regulation of LD content is essential for maintaining cellular homeostasis, as excessive LD accumulation leads to lipotoxicity, organelle dysfunction, and the development of metabolic diseases such as non-alcoholic fatty liver disease and obesity (Mashek, 2021). To prevent such deleterious consequences, cells have evolved tightly regulated mechanisms to degrade LD and release stored fatty acids for energy production or membrane biosynthesis (Zechner et al., 2012). The degradation of LD occurs through two main mechanisms: lipophagy and lipolysis (Corbo and Chung, 2024; Singh et al., 2009; Zechner et al., 2017). Lipophagy is a selective form of autophagy that targets LD for lysosomal degradation and plays a crucial role in cellular metabolism, energy homeostasis, and protection against lipotoxicity (Kounakis et al., 2019). Lipolysis is the enzymatic breakdown of LD into free fatty acids and glycerol, which provide energy and metabolic intermediates for cellular processes (Bolsoni-Lopes and Alonso-Vale, 2015). In immune cells, such as macrophages, lipolysis plays an important role not only in energy homeostasis but also in modulating immune responses and pathogen defense (Leopold Wager et al., 2019; Wang et al., 2019). Recent studies have suggested that the activation of lipolysis can restrict the growth of certain pathogens by depriving them of lipid resources (Vrieling et al., 2019). Therefore, defining how lipolysis is regulated in infected macrophages may clarify whether host lipid metabolism can be therapeutically targeted during hvKp infection. This study aimed to determine whether berberine limits hvKp survival by modulating macrophage inflammatory responses and lipid metabolism, with particular attention to LD turnover, transcription factor EB (TFEB) signaling, autophagy, and lipolysis.

## Materials and Methods

### Cell culture

For all experiments, RAW 264.7 cells (TIB-71, American Type Culture Collection) were maintained at 37℃ in 5% CO_2_ incubator using Dulbecco’s Modified Eagle’s Medium (DMEM; Welgene, Korea) containing 10% fetal bovine serum (Welgene) and 1% antibiotics (Gibco BRL, USA).

### Bacterial strains and culture

Samples obtained from patients diagnosed with liver abscess or pneumonia at Keimyung University Dongsan Hospital (Korea) were used to isolate Kp strains, DSMC-K210, DSMC-K285, and DSMC-K305. Capsular serotypes and virulence-associated genes were analyzed by polymerase chain reaction (PCR). Identification of the isolates and antimicrobial susceptibility profiles were assessed using the VITEK 2 automated system (bioMérieux, France). In addition, extended-spectrum β-lactamase production was determined according to Clinical and Laboratory Standards Institute guidelines using an agar dilution method with cefotaxime, ceftazidime, and ceftazidime-clavulanate. The clinical Kp strains used in this study are listed in Table S1. For preservation, bacteria were stored at –80℃ after addition of 15% (v/v) glycerol (Sigma-Aldrich, USA). To prepare inocula for infection experiments, frozen bacterial stocks were thawed, and Kp strains were cultured overnight in Luria-Bertani (LB) broth at 37℃, followed by subculture in fresh medium until reaching an optical density of approximately 0.1 at 600 nm. Before infection assays, viable counts were determined by measuring colony-forming units (CFUs).

### Infection and treatments

RAW 264.7 cells were seeded into 24-well plates at a density of 2 × 10^5^ cells/well and infected with the indicated Kp strains at a multiplicity of infection (MOI) of 1 or 40 for 1 h. Cells were then washed three times with phosphate-buffered saline (PBS). To eliminate extracellular bacteria, cells were incubated in fresh medium containing 100 μg/ml gentamicin (15710-072, Gibco) for 30 min. The medium was replaced with fresh medium, and berberine (B3251, Sigma-Aldrich) was added following gentamicin treatment and maintained throughout the experiment. To inhibit lipolysis, cells were treated with atglistatin (15284, Cayman Chemical, USA) for 1 h prior to infection, and treatment was maintained throughout the infection period. All cells were incubated at 37°C in 5% CO₂. All treatments were dissolved in dimethyl sulfoxide (DMSO; 20–139, Sigma-Aldrich).

### Cell viability assay

RAW 264.7 cells were plated in 96-well plates at a density of 4 × 10^4^ cells/well and allowed to adhere for 18 h at 37℃ in 5% CO_2_ incubator. Following the treatment with berberine for 6 h, MTS reagent of the CellTiter 96^®^ AQueous One Solution Cell Proliferation Assay kit (MTS; G3580, Promega, USA) was then added to each well and incubated for 30 min at 37℃. Absorbance was recorded at 490 nm using a Synergy/HTX multimode microplate reader (BioTek Instruments, Inc., USA). Relative cell viability was determined against untreated controls set at 100% from three independent experiments performed in triplicate.

### RNA isolation and real-time quantitative PCR (RT-qPCR)

Total RNA was isolated using TRIzol reagent (15596-026, Thermo Fisher Scientific, USA). Complementary DNA (cDNA) was synthesized from the extracted RNA using a reverse transcriptase premix (EBT-1515, Elpis Biotech, Korea). Gene expression analysis by qPCR was conducted using the qTOWER3 PCR thermal cycler (Analytik Jena, Germany) in combination with THUNDERBIRD SYBR qPCR mix (QPS-201, TOYOBO, Japan), and relative expression levels were calculated using the 2^-ΔΔCt^ method and normalized to *β-actin* or *Gapdh*. The mouse primer sequences used in this study are listed in Table S2.

### CFU assay in macrophages

RAW 264.7 cells were infected with hvKp (DSMC-K210 or DSMC-K285, MOI of 40) or cKp (DSMC-K305, MOI of 1) for 1 h at 37℃ in a 5% CO_2_ atmosphere using antibiotic-free medium. To eliminate extracellular bacteria, infected cells were exposed to medium containing 100 µg/ml gentamicin for 30 min after washing with PBS. The cells were then washed and cultured in fresh medium for the indicated time. For intracellular bacterial quantification, cells were lysed with 1% saponin. Following 10-fold serial dilution, the lysates were spread onto LB agar plates, and CFUs were determined after overnight incubation at 37℃.

### Immunoblotting

Cell lysates were prepared using RIPA lysis buffer (89900, Thermo Fisher Scientific), and the lysates were clarified by centrifugation to collect the supernatant fractions. A bicinchoninic acid assay kit (23225, Pierce, USA) was used to determine protein concentrations. After loading equal amounts of protein (50 µg/lane), electrophoresis was performed on sodium dodecyl-sulfate polyacrylamide gels (8–12%), and the separated proteins were transferred to nitrocellulose membranes (GE Healthcare Life Science, USA). Membranes were blocked and incubated with primary antibodies listed in Table S3 overnight at 4℃. After washing, membranes were exposed with appropriate secondary antibodies including anti-rabbit IgG (111-035-045) and anti-mouse lgG (115-035-062) (both diluted 1:5,000; Jackson ImmunoResearch Laboratories, USA). Protein signals were visualized using the Immobilon Western Chemiluminescent HRP Substrate (EMD Millipore, Germany) and captured with a ChemiDoc imaging system (Analytik Jena, Germany).

### Nuclear and cytoplasmic fractionation

RAW 264.7 cells were infected with hvKp (DSMC-K285, MOI 40) and treated with berberine (50 μM) for the indicated times. Cells were harvested and separated into cytoplasmic and nuclear fractions using NE-PER^TM^ Nuclear and Cytoplasmic Extraction Reagents (78833, Thermo Scientific^TM^, USA) according to the manufacturer’s instructions. Equal amounts of protein from each fraction were separated by sodium dodecyl-sulfate polyacrylamide gel electrophoresis and subjected to immunoblotting as described above. TFEB levels were analyzed in both the cytoplasmic and nuclear fractions. ACTB and lamin B1 were used as cytoplasmic and nuclear fraction markers, respectively. The nuclear translocation of TFEB was quantified by calculating the ratio of nuclear TFEB (normalized to lamin B1) to cytoplasmic TFEB (normalized to ACTB).

### Enzyme-linked immunosorbent assay (ELISA)

RAW 264.7 cells were plated in 24-well plates at a density of 2 × 10^5^ cells/well. Culture supernatants were harvested at 4 h and 6 h after incubation and clarified by centrifugation at 300 × g for 5 min. Levels of tumor necrosis factor-alpha (TNF-α; MTA00B-1) and interleukin-6 (IL-6; M6000B-1) were quantified us­ing mouse Quantikine ELISA kits (R&D Systems, USA).

### Immunofluorescence and analysis

The cells grown on coverslips were infected with hvKp and subsequently fixed for 15 min with 4% paraformaldehyde. After fixation, cells were permeabilized for 10 min at room temperature with 0.25% Triton X-100 (Sigma-Aldrich). Samples were then incubated with BODIPY 493/503 (D3922, Molecular Probes, USA), TFEB (BETHYL, Invitrogen), or anti-LC3 antibody (PM036, MBL International) according to the manufacturers’ instructions. Nuclei were counterstained with 4′,6-diamidino-2-phenylindole (DAPI; D9542, Sigma-Aldrich) for 5 min, fluorescence images were captured using a confocal laser-scanning microscope (TCS SP8, Leica Microsystems, Germany) and analyzed using ImageJ software (National Institutes of Health, USA).

### Flow cytometric analysis of LC3

To assess LC3 levels, cells were first fixed for 15 min using 4% paraformaldehyde, followed by permeabilization for 10 min with 0.25% Triton X-100 (Sigma-Aldrich). Cells were then blocked and incubated with an anti-LC3 primary antibody (1:100; #83506, Cell Signaling Technology) for 1 h at room temperature. After washing with PBS, cells were incubated for 1 h at room temperature with Alexa Fluor Plus 488 conjugated goat anti-mouse IgG (H+L) secondary antibody (1:400; A11001, Invitrogen, USA). Nuclei were stained with 4′,6-diamidino-2-phenylindole (DAPI; D9542, Sigma-Aldrich) for 5 min at room temperature. LC3-associated fluorescence signals were subsequently analyzed using BD FACScan^TM^ flow cytometer (BD Biosciences, USA), and mean fluorescence intensity values were calculated using FlowJo software (BD Biosciences, USA).

### TG level measurements

Intracellular TG levels were quantified using a triglyceride quantification kit (DG-TGC100, DoGenBio, Korea). Cell lysates (50 μl) obtained from hvKp-infected RAW 264.7 cells were transferred to a 96-well plate and mixed with 50 μl of TG reaction solution. The mixture was incubated at room temperature for 30 min in the dark. TG levels were then determined through measurement of absorbance at 570 nm using a Synergy/HTX multimode microplate reader (BioTek Instruments, Inc.).

### Transmission electron microscopy

To perform transmission electron microscopy analysis, the samples were fixed on ice for 2 h in 2.5% glutaraldehyde and post-fixed with 1% osmium tetroxide. After washing with PBS, the specimens were dehydrated through graded ethanol and propylene oxide solutions prior to Epon 812 embedding and polymerization at 60°C for 24 h. Sections of 70 nm thickness were mounted on 75-mesh copper grids and double-stained with 4% uranyl acetate and lead citrate. Imaging was conducted using a KBSI Bio-HVEM system (JEM-1400Plus at 120 kV and JEM-1000BEF at 1000 kV; JEOL, Japan).

### Free glycerol quantification

To quantify free glycerol, cell culture supernatants were first centrifuged at 3,000 rpm for 3 min, and the clarified fractions were subsequently collected. These samples were then reacted with reagents from the Free Glycerol Kit (ab65337, Abcam, UK) in a 96-well plate for 30 min at room temperature in the dark. The absorbance at 570 nm was measured using a Synergy/HTX multimode microplate reader (BioTek Instruments, Inc.).

### Zebrafish (ZF) husbandry

Wild-type *Danio rerio* (AB strain) was provided by the Korea Zebrafish Resource Center. The fish were maintained in an in-house ZF facility under a 14:10 h light/dark cycle. They received brine shrimp twice daily, with an additional feeding on the day before mating. After natural spawning, the embryos were collected in fish water prepared by dissolving 1 g of Instant Ocean sea salt in 1 L of dH_2_O, and the pH was adjusted to 7.0 using NaHCO_3_ (Takaki et al., 2013). The fertilized eggs were raised in Petri dishes for up to 3 days post-fertilization (dpf), with daily water changes. All ZF experiments were conducted under approval of the Animal Research Ethics Committee of Gyeongsang National University (Approval No. GNU-190325-E0014).

### Preparation of bacterial inoculum

The infection stock of the hvKp strain expressing green fluorescent protein (GFP) was prepared as previously described (Nguyen et al., 2023). Briefly, the strain was inoculated into LB broth supplemented with kanamycin (50 µg/ml) and cultured to mid-logarithmic phase (OD_600_ ≈ 0.8). The cell pellet was harvested by centrifugation at 4,000 rpm for 5 min and washed twice with LB broth. To ensure a uniform suspension, the bacterial cells were passed through a 26-gauge needle and divided into 200 µl aliquots for storage at –80°C. To determine a uniform stock concentration, three random tubes were selected, and serial dilutions (10-fold) were plated on LB agar plates containing kanamycin (50 µg/ml). Before injection, the frozen aliquot was thawed, washed twice, and resuspended in PBS to achieve the desired concentration (Marcoleta et al., 2018).

### Microinjection of hvKp in ZF larvae through the caudal vein

ZF embryos were dechorionated between 30–48 h post-fertilization. Further, tricaine stock solution was prepared by dissolving 0.4 g of tricaine in 100 ml dH_2_O with pH adjusted to 7.0 using NaHCO_3_ and stored at 4℃. Prior to use, 630 µl of tricaine stock was diluted in 10 ml of sterilized fish water, yielding a final concentration of approximately 0.24–0.25 mg/ml (Takaki et al., 2013). ZF larvae were anesthetized and mounted on 1.7% agarose prepared with sterilized fish water. A small volume of tricaine solution was added to the plate to maintain anesthesia throughout the injection. Based on prior optimization, an inoculum of ~5,000 CFU/larva was sufficient to establish infection. Accordingly, ~3 nl of the bacterial suspension (5,000 CFU) was injected via the caudal vein using a Tritech Research Digital microinjector (MINJ-D, Tritech Research, USA). Control larvae were injected with an equivalent volume of sterile PBS to exclude nonspecific effects (Ye et al., 2025).

The injection needles were replaced every 100 larvae or 1 h, whichever occurred first. Consistency of the injection was checked by injecting into sterile PBS and plating on LB agar containing 50 µg/ml kanamycin. Post-injection, larvae were transferred to 96-well plates (2 fish/well) and exposed to the drug solutions (Berberine at 3 or 6 µM). Amikacin (AMK; 25 µM) was used as a positive control. A non-infected DMSO-only group was included separately as a vehicle control to monitor possible DMSO toxicity. The drug solution and fish water were changed daily.

### Drug efficacy assessment in hvKp-infected ZF

The in vivo efficacy of berberine was determined by hvKp proliferation (GFP fluorescence intensity within the ZF), larval survival, and bacterial burden after homogenization of ZF larvae. Bacterial proliferation was monitored daily by tracking GFP fluorescence in the infected larvae using the ImageXpress Pico Automated Cell Imaging System (Molecular Devices). The end-point GFP signal intensity was used for comparative analysis. Ten larvae from each treatment group were randomly selected and individually lysed in 2% Triton X-100 in PBS with Tween-20 (Jeon et al., 2022). Samples were homogenized using a handheld homogenizer (D1,000; Benchmark), serially diluted (10-fold), and plated on LB agar containing kanamycin (50 µg/ml). Colonies were counted after an overnight incubation. Survival was monitored daily, and the number of dead larvae in each treatment group was recorded for survival curve analysis.

### Statistical analysis

The Mean ± standard deviation was used to present the data obtained from three independent experiments. To evaluate statistical differences, we used Student’s *t*-test or analysis of variance, depending on the nature of the comparison. GraphPad Prism 7.0 for Windows (GraphPad Software, Inc., USA) was used for all statistical analyses and figure generation, and significance was assigned at different thresholds, including *P* values below 0.05, 0.005, and 0.001.

## Results

### Berberine suppresses intracellular survival of hvKp

Berberine, an isoquinoline alkaloid (Fig. 1A), regulates macrophage activation and promotes autophagy (Li et al., 2022; Zheng et al., 2021). To evaluate its cytotoxicity prior to functional assays, RAW 264.7 cells were treated with berberine and cell viability was assessed. Berberine treatment did not significantly affect macrophage viability compared with the untreated control (Fig. 1B). To determine whether berberine directly affects bacterial growth, we used two hvKp strains (DSMC-K210 or DSMC-K285) isolated from patients (Jung et al., 2024). Co-treatment with berberine and hvKp did not affect cell viability, indicating that berberine is non-toxic, even during infection (Fig. 1C). Berberine did not affect bacterial growth under these conditions, as no significant differences were observed between the groups (Fig. S1). To evaluate the effects of berberine on intracellular bacterial survival, murine macrophages were infected with hvKp and treated with berberine. A significant reduction in the intracellular bacterial load was observed in the berberine-treated group compared with that in the untreated control group (Fig. 1D). To evaluate the antibacterial effects of berberine in vivo, ZF larvae were infected with GFP-expressing hvKp and treated with berberine. Berberine treatment markedly decreased the GFP fluorescence intensity of hvKp in ZF larvae compared with that in the untreated group, indicating a decreased bacterial burden (Fig. 1E). Berberine treatment significantly reduced bacterial CFU counts in infected ZF embryos (Fig. 1F). Moreover, ZF survival analysis demonstrated that berberine treatment markedly improved host survival compared with that of the infection-only group (Fig. 1G). Similar to the positive control AMK, berberine exhibited protective effects against hvKp infection in vivo. These findings indicate that berberine suppresses intracellular hvKp survival both in vitro and in vivo. Given that berberine did not directly inhibit bacterial growth (Fig. S1) but significantly reduced the intracellular bacterial load in infected macrophages (Fig. 1D), these data suggest that berberine acts through host cell-associated mechanisms rather than through direct antibacterial activity.

### Berberine attenuates hvKp-induced inflammatory responses and mitogen-activated protein kinase signaling

Berberine is known to exert anti-inflammatory effects on macrophages (Zhang et al., 2023). To determine whether berberine similarly attenuates inflammatory responses during hvKp infection, murine macrophages were infected with hvKp strains and treated with berberine. Berberine significantly reduced the mRNA levels of inflammatory cytokines, including interleukin *(Il)6*, tumor necrosis factor *(Tnf)α*, *Il1b*, and *Il18*, which were elevated following hvKp infection (Fig. 2A). Berberine treatment markedly decreased the secretion of IL-6 and TNF-α in the supernatants of hvKp-infected macrophages (Fig. 2B). In macrophages, inflammatory cytokine expression is regulated by the activation of mitogen-activated protein kinase (MAPK) pathway proteins, including extracellular signal-regulated kinase (ERK) and c-Jun N-terminal kinase (JNK) (Tian et al., 2019). As Kp infection in macrophages is known to increase the phosphorylation of the MAPK pathway proteins ERK and JNK (March et al., 2011; Wu et al., 2006; Xu et al., 2017), we next examined whether hvKp infection induces ERK and JNK phosphorylation and whether berberine co-treatment affects this process. We found that hvKp infection markedly increased ERK and JNK phosphorylation, and that this increase was significantly attenuated by berberine co-treatment (Fig. 2C). These findings indicate that berberine effectively suppresses hvKp-induced inflammatory responses and ERK/JNK phosphorylation in macrophages.

### Berberine inhibits LD formation in hvKp-infected murine macrophages

Previous studies have shown that LD formation is crucial for the survival of hvKp within macrophages (Jung et al., 2024). Given that the mammalian target of rapamycin (mTOR) signaling regulates lipid metabolism and contributes to macrophage host defense against Kp infection (Han and Wang, 2018; Jung et al., 2024), we investigated whether berberine modulates LD formation during hvKp infection via the mTOR pathway (Fig. 3A). Infection with hvKp significantly increased the phosphorylation of mTOR and its downstream effectors p70S6K and S6 compared with the uninfected control group. Berberine treatment markedly suppressed hvKp-induced phosphorylation of mTOR, p70S6K, and S6, suggesting that berberine inhibits the mTOR/p70S6K/S6 signaling cascade activated by hvKp infection. As mTOR signaling is known to promote lipogenic gene expression (Düvel et al., 2010), we next assessed the expression of key lipogenesis-related genes, including fatty acid synthase (*Fasn*), stearoyl-CoA desaturase 1 (*Scd1*), and acetyl-CoA carboxylase alpha (*Acaca*). The mRNA levels of these genes were upregulated following hvKp infection, but were markedly reduced by berberine treatment (Fig. 3B). Berberine treatment significantly reduced hvKp-induced LD accumulation, as visualized by confocal microscopy (Fig. 3C). To investigate the changes in LD during hvKp infection and berberine treatment, we examined the expression of perilipin 1 (PLIN1), a major protein localized on the LD membrane. Berberine significantly attenuated the infection-induced upregulation of PLIN1 (Fig. 3D), further supporting that berberine suppresses hvKp-induced LD formation. Furthermore, the quantification of intracellular TG levels demonstrated a marked increase following hvKp infection, which was significantly reduced by berberine co-treatment (Fig. 3E). Transmission electron microscopy further confirmed that hvKp-induced LD formation was attenuated by berberine treatment, validating our observations at the ultrastructural level (Fig. 3F). Collectively, these results demonstrate that berberine attenuates hvKp-induced lipogenesis and LD accumulation in macrophages, at least in part through inhibition of the mTOR/p70S6K/S6 signaling pathway.

### Berberine promotes activation and nuclear translocation of transcription factor EB during hvKp infection

TFEB is a master regulator of autophagy and lysosomal biogenesis, and has been implicated in the regulation of lipophagy and lipolysis (Li et al., 2025b). Moreover, berberine modulates the activation of TFEB in macrophages (Zheng et al., 2021). As berberine decreased LD formation in hvKp-infected macrophages, we investigated whether TFEB signaling was involved in this process. To this end, we measured the mRNA levels of autophagy-related genes, including microtubule-associated protein 1 light chain 3 beta (LC3; encoded by *Map1lc3b*), sequestosome 1 (p62; encoded by *Sqstm1*), and GABA type A receptor-associated protein (*Gabarap*). Berberine co-treatment significantly increased the mRNA expression of these autophagy-related genes in hvKp-infected RAW 264.7 cells (Fig. 4A). We next evaluated *Tfeb* mRNA expression following hvKp infection and found that *Tfeb* expression levels were markedly decreased 2 h post-infection and were restored by berberine treatment (Fig. 4B). To determine whether berberine promotes TFEB nuclear translocation, we performed nuclear and cytoplasmic fractionation to assess the subcellular distribution of TFEB. Berberine treatment enhanced TFEB nuclear accumulation in hvKp-infected murine macrophages, as shown by fractionation and imaging analyses (Fig. 4C–4D). Collectively, these data demonstrate that berberine promotes TFEB activation, as evidenced by its enhanced nuclear translocation, in hvKp-infected murine macrophages.

### Berberine does not restore hvKp-induced suppression of autophagy and autophagic flux in macrophages

Berberine has been shown to reduce LD in various cell types by modulating lipid metabolic pathways (Ren et al., 2020; Wang et al., 2025). Here, we investigated whether berberine removes LD through autophagy activation. We examined the protein levels of LC3, a key autophagy-related protein, and found that LC3 levels were not significantly increased in hvKp-infected murine macrophages compared with those in uninfected controls, suggesting that autophagy was not activated during infection (Fig. 5A). To determine whether this lack of autophagy induction was specific to hvKp, we compared LC3 expression between hvKp- and cKp-infected macrophages. In contrast to hvKp infection, LC3 expression in cKp-infected macrophages gradually increased over time (Fig. 5B). Confocal microscopy confirmed that LC3 expression was significantly higher in cKp-infected macrophages than in hvKp-infected macrophages (Fig. 5C). Berberine treatment failed to induce LC3 expression in hvKp-infected murine macrophages (Fig. 5D). To further assess autophagic flux, we examined p62 levels and confirmed p62 protein levels did not decrease over time in either the hvKp-infected or berberine co-treated groups, indicating that autophagic flux remained impaired and was not functionally restored by berberine (Fig. 5E). Collectively, these results indicate that hvKp, unlike cKp, suppresses autophagy induction and impairs autophagic flux in macrophages, and that berberine co-treatment is insufficient to restore functional autophagy under these conditions.

### Berberine enhances lipolysis in hvKp-infected murine macrophages

To investigate the mechanism by which berberine regulates lipid metabolism during hvKp infection, we focused on the lipolysis pathway and measured the mRNA levels of perilipin 2 (PLIN2; encoded by *Plin2*), a LD coat protein that restricts lipase access to LD, along with two key lipolytic enzymes: hormone-sensitive lipase (HSL; encoded by *Lipe*) and adipose triglyceride lipase (ATGL; encoded by *Pnpla2*). Compared with hvKp-infected cells, berberine-treated cells showed a significant increase in the mRNA expression of *Pnpla2* and *Lipe*. In contrast, the expression of *Plin2* was markedly decreased (Fig. 6A). Berberine treatment restored ATGL protein expression, which was reduced by hvKp infection (Fig. 6B). Berberine treatment also increased the phosphorylation of HSL at Ser563 and Ser660, indicating activation of the lipolytic enzyme cascade (Fig. 6B). Berberine treatment increased the level of free glycerol compared with hvKp infection alone, indicating enhanced TG breakdown through lipolysis (Fig. 6C). To determine whether ATGL-mediated lipolysis is required for the protective effect of berberine, we co-treated hvKp-infected macrophages with atglistatin, a selective ATGL inhibitor. Atglistatin treatment abolished the berberine-induced reduction in intracellular bacterial survival, indicating that ATGL-dependent lipolysis is required for berberine-mediated suppression of hvKp survival (Fig. 6D). Collectively, these results indicate that berberine promotes lipolysis, rather than lipophagy, as the primary mechanism through which it modulates lipid metabolism and suppresses LD accumulation during hvKp infection.

## Discussion

Berberine is a plant-derived isoquinoline alkaloid extracted from *Berberis vulgaris* (barberry), *Coptis chinensis* (Chinese goldthread), and *Hydrastis canadensis* (goldenseal) (Cicero and Baggioni, 2016). It exhibits diverse pharmacological activities, including anti-inflammatory, antimicrobial, and metabolic regulatory effects (Gasmi et al., 2024). In a mouse model of *Salmonella enterica* serovar Typhimurium infection, berberine alleviated enteritis and protected against endotoxic shock (Li et al., 2022). Berberine exhibits significant antimicrobial activity against methicillin-resistant *Staphylococcus aureus* isolated from patients with bloodstream infections (Xia et al., 2022). Although its protective effects have been demonstrated in several infectious disease models, its role in host metabolic regulation during hvKp infection has not yet been defined. In this study, we demonstrated that berberine significantly reduced intracellular hvKp survival in macrophages and modulated infection-associated lipid metabolic remodeling. These findings provide mechanistic insights into how host-directed metabolic intervention may influence hvKp pathogenesis.

Accumulating evidence indicates that metabolic reprogramming is a critical determinant of host–pathogen interactions (Gleeson and Sheedy, 2016; O'Neill and Pearce, 2016). LDs, once considered inert lipid storage organelles, are now recognized as dynamic regulators of immune responses and intracellular pathogen survival. For instance, in *Mycobacterium tuberculosis* infection, restricting fatty acid uptake by macrophages limits bacterial growth (Simwela et al., 2025). Similarly, during hvKp infection, LD formation functions as an energy reservoir and plays a critical role in supporting the survival of intracellular bacteria (Jung et al., 2024). However, the mechanisms by which host lipid metabolism can be therapeutically targeted to restrict hvKp survival remain poorly understood. In the present study, we demonstrated that berberine markedly suppressed LD accumulation in hvKp-infected macrophages, thereby limiting intracellular bacterial survival. Mechanistically, our findings suggest that berberine restricts hvKp replication by altering host lipid metabolism and reducing lipid availability in infected macrophages.

LD turnover in macrophages can be regulated through two major pathways: lipophagy and lipolysis. Lipophagy is the selective autophagic degradation of LD via lysosomal pathways and plays a crucial role in maintaining cellular lipid homeostasis and energy balance (Khawar et al., 2019). Lipolysis is an enzymatic breakdown process in which TGs are hydrolyzed into free fatty acids and glycerol (Lass et al., 2011). Previous studies have shown that Kp infection impairs autophagic responses in macrophages (Wang et al., 2023), and berberine has been reported to regulate sirtuin 3-mediated lipophagy (Chen et al., 2025). Based on these observations, we initially hypothesized that berberine reduces LD accumulation by restoring lipophagy during hvKp infection. Consistent with this possibility, berberine treatment increased the expression of autophagy-related genes. TFEB, a key transcription factor that promotes autophagy (Zheng et al., 2021), was suppressed during hvKp infection but was restored following berberine treatment. Despite these transcriptional changes, autophagic flux remained largely unchanged, indicating that lipophagy was not functionally restored under these conditions. This unexpected finding suggests that LD reduction during hvKp infection occurs independently of lipophagy. Instead, our results suggest an alternative mechanism involving lipolysis. Supporting this interpretation, previous studies have shown that TFEB can regulate lipolytic pathways in addition to lipophagy (Li et al., 2021) and that berberine induces lipolysis in adipocytes by activating the AMP-activated protein kinase (AMPK) pathway (Yang et al., 2020). Taken together, our findings reveal that berberine limits intracellular hvKp survival by promoting lipolysis rather than lipophagy, thereby reducing the host lipid availability required for bacterial persistence. These results highlight the previously underappreciated role of lipolysis in controlling infection-induced LD accumulation, and suggest that targeting host lipid metabolism may represent a potential host-directed therapeutic strategy against hvKp infection.

Additionally, inflammatory responses play a crucial role in host defense by promoting pathogen clearance (Cronkite and Strutt, 2018). Excessive inflammation can lead to tissue damage (Paludan et al., 2021). HvKp infection triggers robust inflammatory responses in macrophages by activating the spleen tyrosine kinase-dependent immune-responsive gene 1-itaconate pathway (Mina et al., 2024) and inducing mitochondrial damage that promotes NOD-, LRR-, and pyrin domain-containing protein 3 inflammasome activation (Li et al., 2025a), highlighting the importance of maintaining homeostasis. Previous studies have shown that berberine exerts anti-inflammatory effects on macrophages (Liu et al., 2018; Xiong et al., 2023). Consistent with these reports, we observed that berberine effectively attenuated hvKp-induced inflammatory responses. Berberine is known to modulate MAPK signaling in macrophages under inflammatory conditions by activating AMPK (Jeong et al., 2009). Our findings indicate that berberine suppresses the activation of the JNK and ERK pathways induced by hvKp infection. This inhibition likely reduces pro-inflammatory cytokine production, limits excessive immune-mediated tissue damage, and preserves host defense.

Berberine has been reported to reduce intracellular lipid accumulation and oxidative stress through modulation of Lrrc58a in zebrafish hepatocytes (Wang et al., 2026), and a similar lipid-regulatory mechanism may contribute to the restoration of macrophage bactericidal function observed in our in vitro model. In the zebrafish system, berberine has likewise been shown to suppress macrophage and neutrophil recruitment and to downregulate TNF-α, IL-1β, and IL-6 expression (Zhang et al., 2020). Given the evolutionary conservation of NF-κB and related inflammatory signaling pathways, these findings collectively suggest that berberine may exert its protective effects through a conserved innate immune axis across both experimental systems.

Several limitations should be considered. First, this study was conducted in RAW 264.7 murine macrophages, which may not fully represent primary or tissue-resident macrophage responses. Second, although hvKp infection was associated with reduced *Tfeb* expression and impaired autophagy-related responses, the upstream signaling mechanisms remain to be clarified. Third, additional mechanistic in vivo validation is required to determine whether berberine-mediated lipolysis restricts bacterial survival during systemic infection. Despite these limitations, our results highlight that lipid metabolic regulation is a key determinant of hvKp intracellular survival. By promoting lipolysis and attenuating MAPK-driven inflammation, berberine disrupts the metabolic conditions favorable for bacterial persistence while moderating excessive host responses. These findings support further investigation of berberine as a potential host-directed therapeutic strategy targeting the metabolic pathways involved in hvKp pathogenesis.

## Figures and Tables

**Fig. 1.**
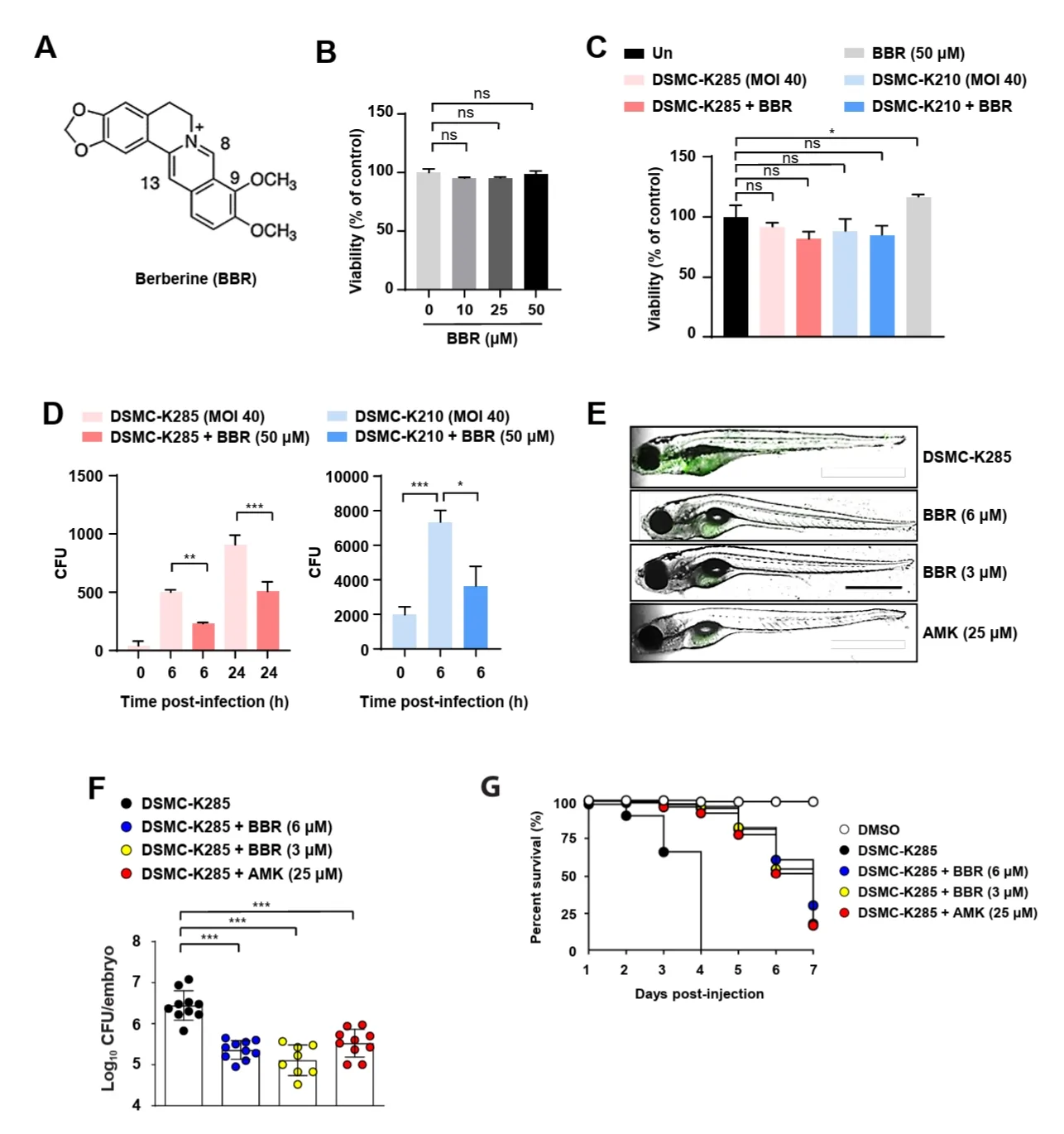
Berberine inhibits intracellular hvKp survival. (A) Chemical structure of berberine. (B) RAW 264.7 cells were treated with berberine (10, 25, or 50 μM) for 6 h, and cell viability was assessed using the MTS assay. (C) RAW 264.7 cells were infected with hvKp (DSMC-K210 or DSMC-K285, MOI 40) and then treated with berberine (50 μM) for 6 h. Cell viability was subsequently evaluated using the MTS assay. (D) RAW 264.7 cells were infected with hvKp (DSMC-K210 or DSMC-K285, MOI 40) and treated with berberine (50 μM) for the indicated times. Intracellular bacterial burden was measured using the CFU assay. (E) Representative images of GFP-expressing hvKp infection in ZF larvae. Fluorescence intensity was reduced in larvae treated with berberine or AMK compared with the untreated infected group. (F) Bacterial burden (log_10_ CFU/larva) in hvKp-infected ZF larvae treated with berberine or AMK. (G) Kaplan–Meier survival curves of hvKp-infected ZF larvae treated with berberine or AMK. Zebrafish larvae were infected at 3 days post-fertilization (dpf), followed by antibiotic treatment, and survival was monitored for 7 days post-injection (dpi). Survival was prolonged in the berberine- and AMK-treated groups compared with the untreated infected group. Data in panels B–D are expressed as the Mean ± SD from three independent experiments (n = 3). Statistical significance was determined using Student’s *t*-test. *P < 0.05, **P < 0.005, ***P < 0.001. Data in panel F are presented as the Mean ± SEM (n = 10 larvae/group). Statistical significance was determined by one-way ANOVA followed by Dunnett’s multiple comparisons test, with p-values calculated relative to the untreated infected control group (Un), where ***P < 0.001. Un, untreated (control group); ns, not significant; AMK, amikacin; BBR, berberine.

**Fig. 2.**
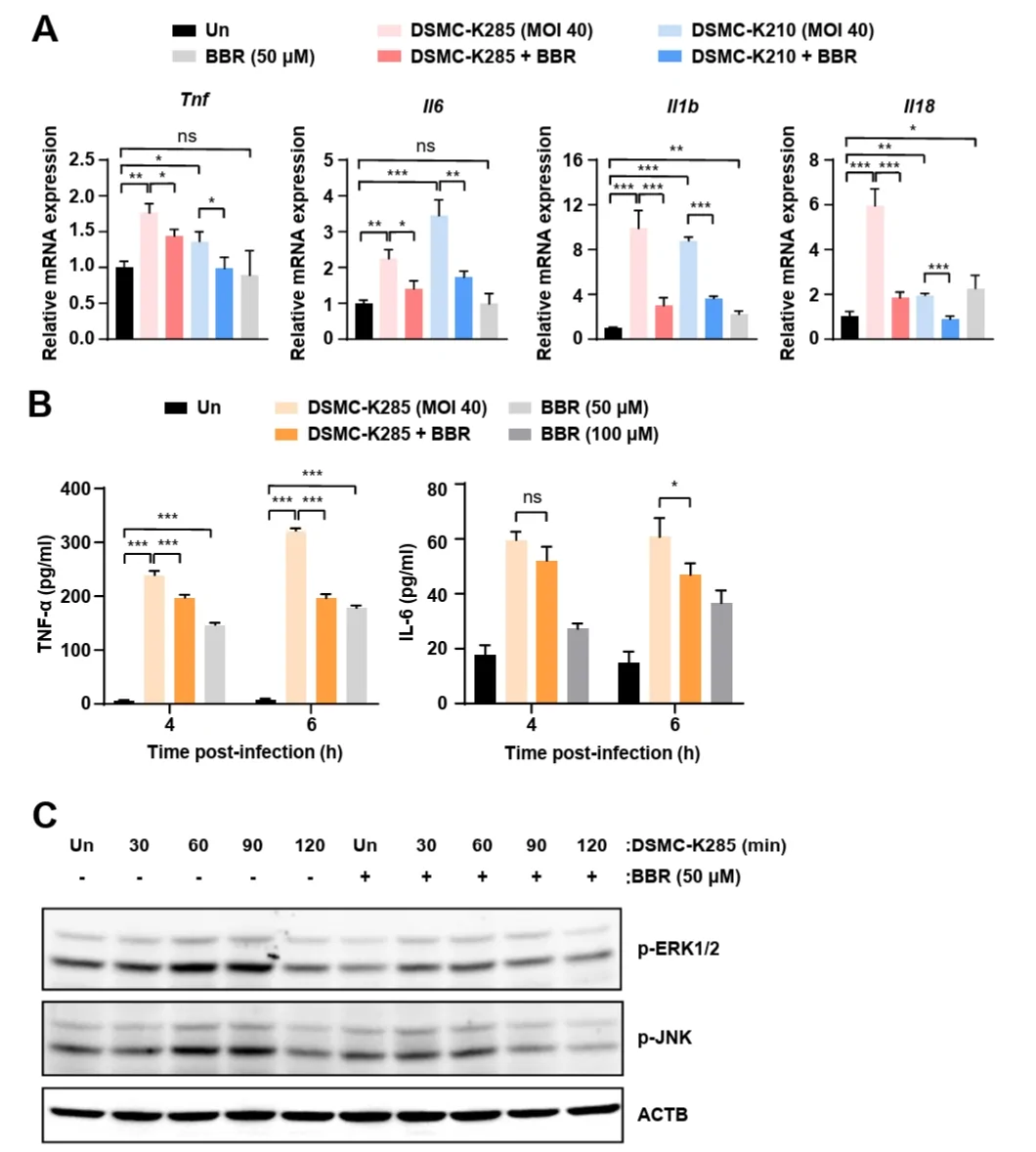
Berberine reduces inflammation in hvKp-infected macrophages. (A) RAW 264.7 cells infected with hvKp (DSMC-K210 or DSMC-K285, MOI 40) were treated with berberine (50 μM) for 4 h, and pro-inflammatory cytokine mRNA levels were measured by RT-qPCR. (B) RAW 264.7 cells infected with hvKp (DSMC-K285, MOI 40) were treated with berberine (50 or 100 μM), and protein levels of TNF-α and IL-6 in the culture supernatants were measured by ELISA. (C) RAW 264.7 cells were infected with hvKp (DSMC-K285, MOI 40), treated with berberine (50 μM), and harvested at the indicated time points for Western blot analysis of ERK and JNK phosphorylation. ACTB was used as the loading control. All quantitative data are expressed as the Mean ± SD from three independent experiments (n = 3). Statistical significance was determined using Student’s *t*-test. *P < 0.05, **P < 0.005, ***P < 0.001. Un, untreated (control group); ns, not significant; BBR, berberine.

**Fig. 3.**
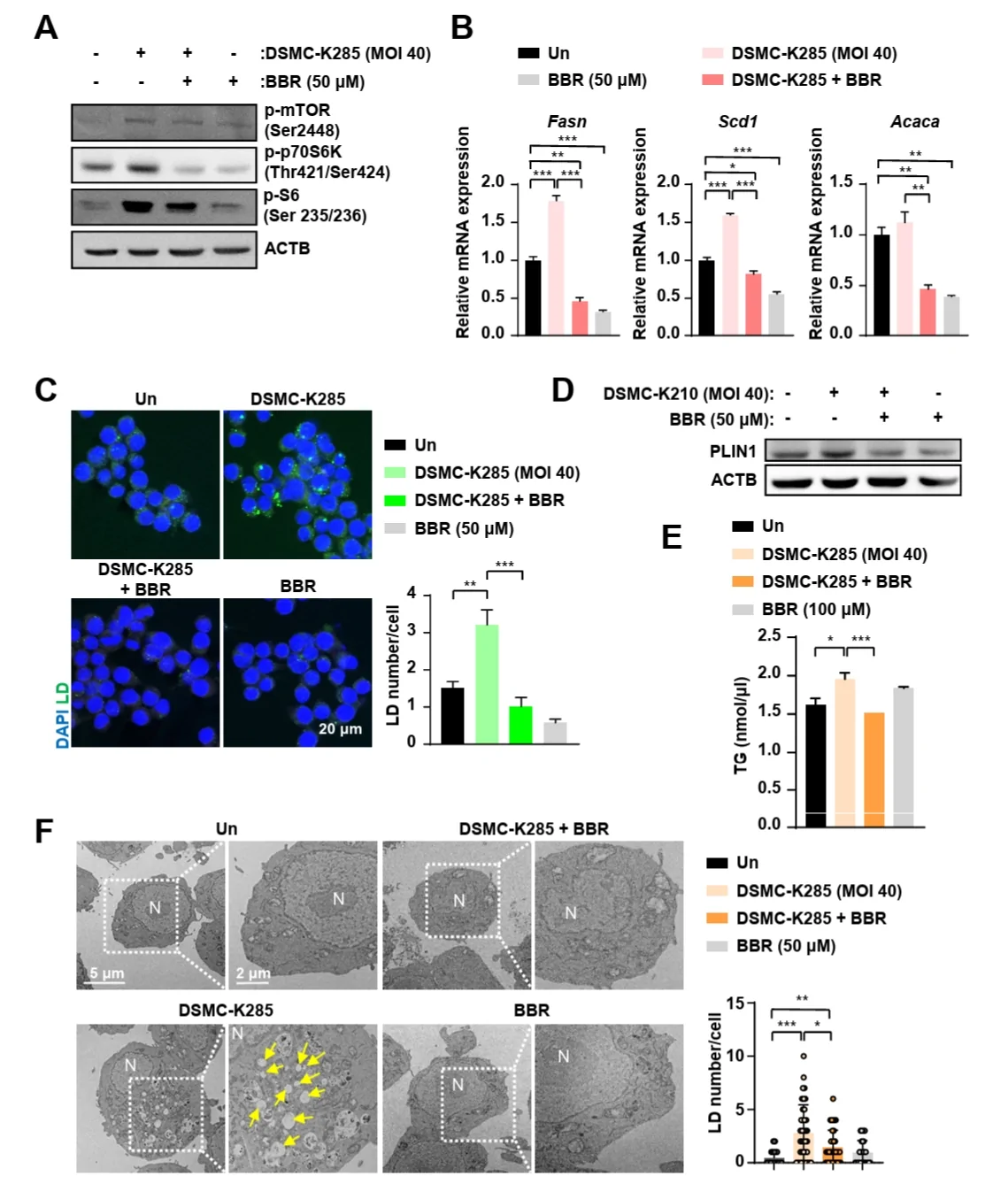
Berberine reduces hvKp-induced LD accumulation in macrophages. (A) RAW 264.7 cells were infected with hvKp (DSMC-K285, MOI 40) and treated with berberine (50 μM) for 6 h, and mTOR signaling was assessed by Western blot. ACTB was used as the loading control. (B) RAW 264.7 cells were infected with hvKp (DSMC-K285, MOI 40) and treated with berberine (50 μM) for 4 h. The expression levels of lipogenesis-related genes (*Fasn*, *Scd1*, and *Acaca*) were analyzed using RT-qPCR. (C) LD formation in RAW 264.7 cells under hvKp infection and berberine treatment was visualized by confocal microscopy. Images are representative of three independent experiments. Scale bar = 20 μm. (D) RAW 264.7 cells were infected with hvKp (DSMC-K210) and treated with berberine (50 μM), and PLIN1 expression was assessed by Western blotting. ACTB was used as the loading control. (E) Intracellular TG levels were quantified in RAW 264.7 cells following hvKp (DSMC-K285) infection and berberine treatment (100 μM). (F) LD in RAW 264.7 cells under hvKp infection and berberine treatment were visualized by TEM and quantified as shown in the panel. Yellow arrows indicate LD. Scale bars = 2 and 5 μm. All quantitative data are expressed as the Mean ± SD from three independent experiments (n = 3). Data in panel F are expressed as the Mean ± SD (n = 30). Statistical significance was determined using Student’s *t*-test. *P < 0.05, **P < 0.005, ***P < 0.001. Un, uninfected (control group); LD, lipid droplet; TG, triglyceride; N, nucleus; BBR, berberine.

**Fig. 4.**
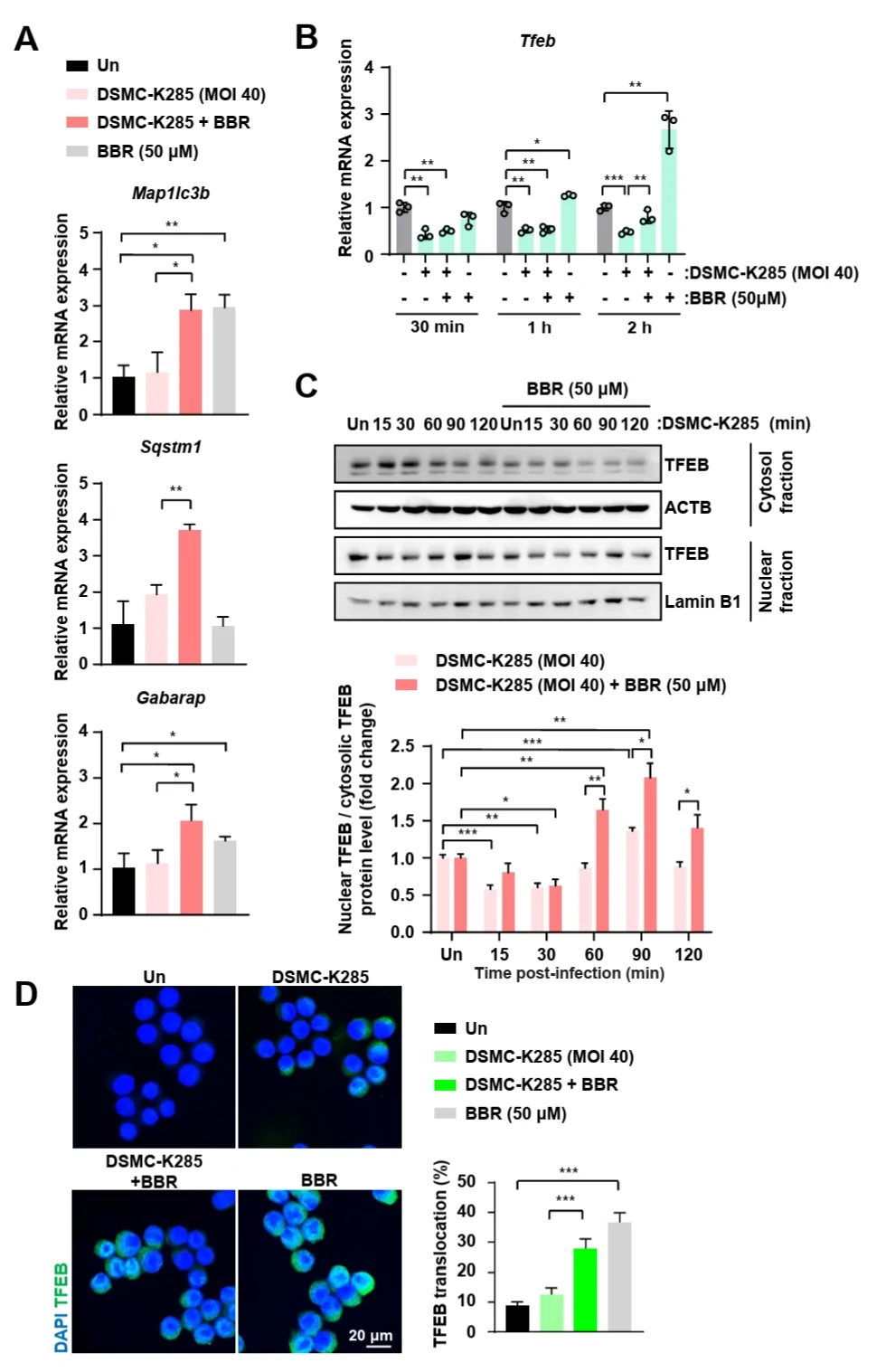
Berberine increases TFEB expression and nuclear accumulation in hvKp-infected macrophages. (A) RAW 264.7 cells were infected with hvKp (DSMC-K285) and treated with berberine (50 μM) for 4 h, and autophagy-related gene expression was measured by RT-qPCR. (B) HvKp-infected RAW 264.7 cells were treated with berberine (50 μM), and *Tfeb* mRNA levels were analyzed by RT-qPCR at the indicated time points. (C) TFEB protein levels in nuclear and cytosolic fractions were assessed by Western blotting after berberine treatment at the indicated time points. ACTB and lamin B1 were used as cytosolic and nuclear fraction controls, respectively. (D) Confocal microscopy was performed to visualize the nuclear translocation of TFEB following berberine treatment. Images are representative of three independent experiments. Scale bar = 20 μm. Data are expressed as the Mean ± SD from three independent experiments (n = 3). Statistical significance was determined using Student’s *t*-test. *P < 0.05, **P < 0.005, ***P < 0.001. Un, uninfected (control group); BBR, berberine.

**Fig. 5.**
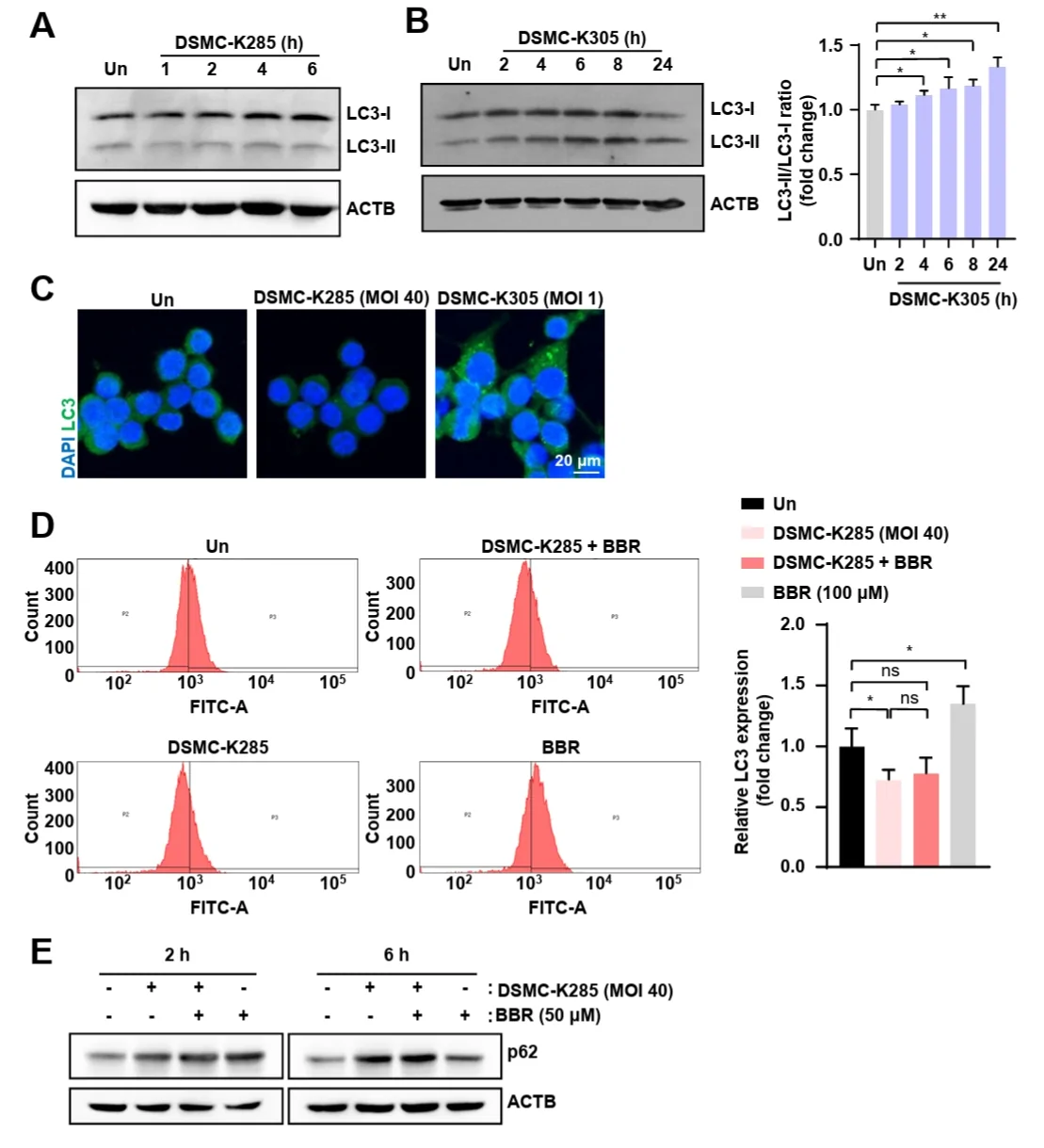
Berberine co-treatment fails to restore autophagy induction and autophagic flux impaired by hvKp infection in macrophages. (A–C) RAW 264.7 cells were infected with hvKp (DSMC-K285, MOI 40) or cKp (DSMC-K305, MOI 1). (A and B) LC3 protein expression was analyzed by Western blotting using ACTB as the loading control. (C) Representative confocal images showing LC3 expression. Scale bar = 20 μm. (D) RAW 264.7 cells infected with hvKp (DSMC-K285) and treated with berberine (100 μM) were analyzed for LC3 fluorescence by flow cytometry. (E) RAW 264.7 cells infected with hvKp (DSMC-K285, MOI 40) and treated with berberine (50 μM). p62 expression was analyzed by Western blot at 2 and 6 h post-infection. Images are representative of three independent experiments. Data are expressed as the Mean ± SD from three independent experiments (n = 3). Statistical significance was determined using Student’s *t*-test. *P < 0.05, **P < 0.005, ***P < 0.001. Un, uninfected (control group); ns, not significant; BBR, berberine.

**Fig. 6.**
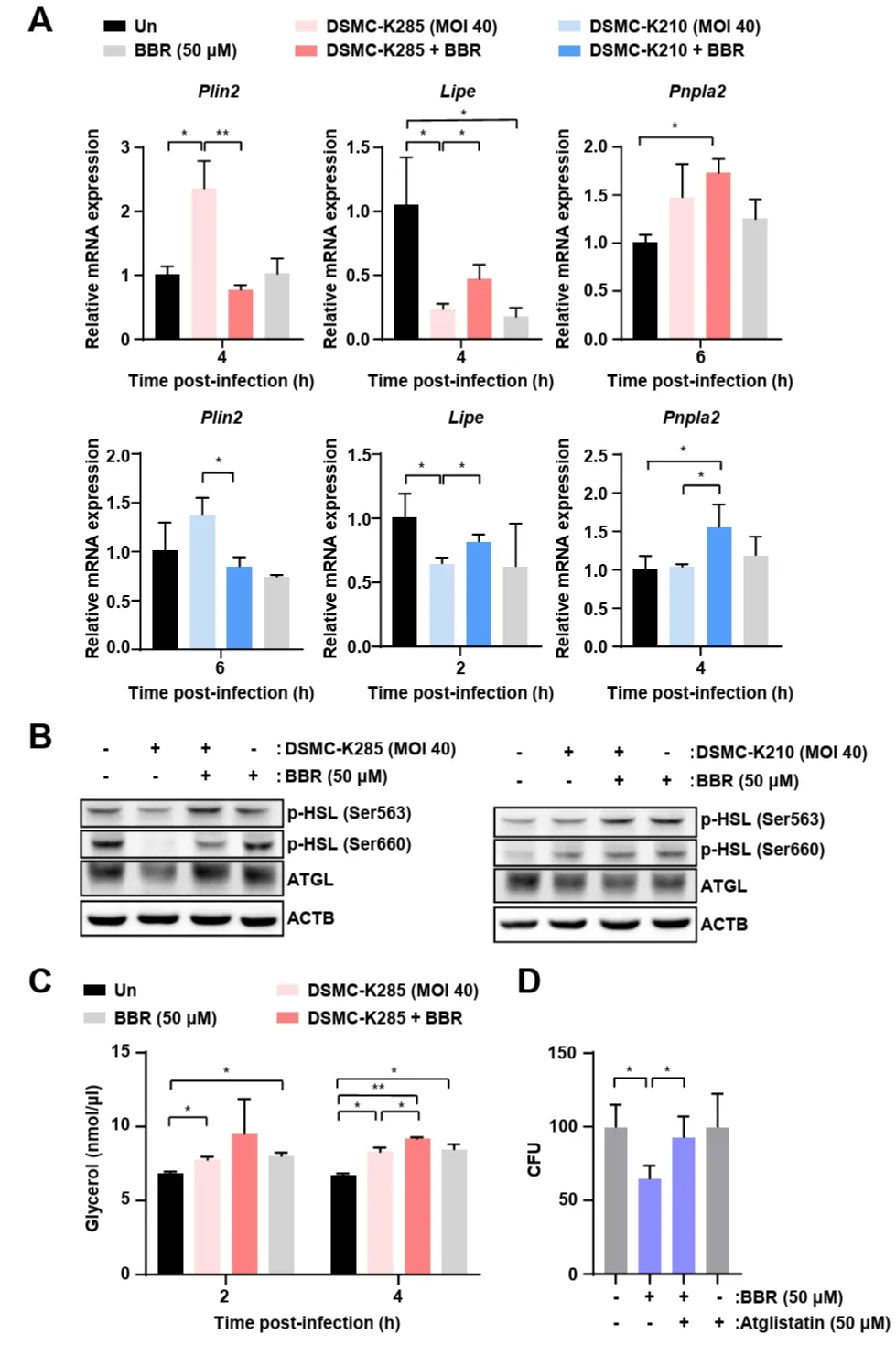
Berberine promotes lipolysis-associated responses in hvKp-infected macrophages. (A) RAW 264.7 cells were infected with hvKp (DSMC-K210 or DSMC-K285, MOI 40) and treated with berberine (50 μM), and the mRNA levels of lipolysis-related genes were measured by RT-qPCR. (B) RAW 264.7 cells were infected with hvKp (DSMC-K210 or DSMC-K285, MOI 40) and treated with berberine (50 μM), and the protein levels of lipolysis-related proteins were measured by Western blot. (C) RAW 264.7 cells were infected with hvKp (DSMC-K285, MOI 40) and treated with berberine (50 μM), and free glycerol levels in the culture supernatant were quantified. (D) RAW 264.7 cells were infected with hvKp (DSMC-K285, MOI 40) and treated with berberine (50 μM) in the presence or absence of the ATGL inhibitor atglistatin (50 μM), and intracellular bacterial burden was measured after 24 h by CFU assay. All data are expressed as the Mean ± SD from three independent experiments (n = 3). Statistical significance was determined using Student’s *t*-test. *P < 0.05, **P < 0.005, ***P < 0.001. Un, untreated (control group); BBR, berberine.

## Supplementary Information

Table S1. Characteristics of Kp strains using in this work

Table S2. Primers used in this study

Table S3. The information on antibodies

Fig. S1. Berberine treatment shows no effect on hvKp growth. HvKp (DSMC-K285 or DSMCK210, MOI 40) were incubated with berberine (50 or 100 μM) at various time points in culture medium. All data expressed as the Mean ± SD from three independent experiments (n = 3). Statistical significance was determined using Student’s *t*-test. ^*^ *P* < 0.05, ^**^ *P* < 0.005, and ^***^ *P* < 0.001 according to analysis of variance. ns, not significant; BBR, berberine.
