# Supplementary material for: Berberine promotes host lipolysis to enhance antimicrobial defense against hypervirulent Klebsiella pneumoniae infection
Source: J Microbiol. 2026 Aug 31;64(8):e2605001. doi: 10.71150/jm.2605001 (PMC13688129; doi:10.71150/jm.2605001)
Supplement: Supplementary file 1 [file jm-2605001-Supplementary-Fig-S1.pdf]

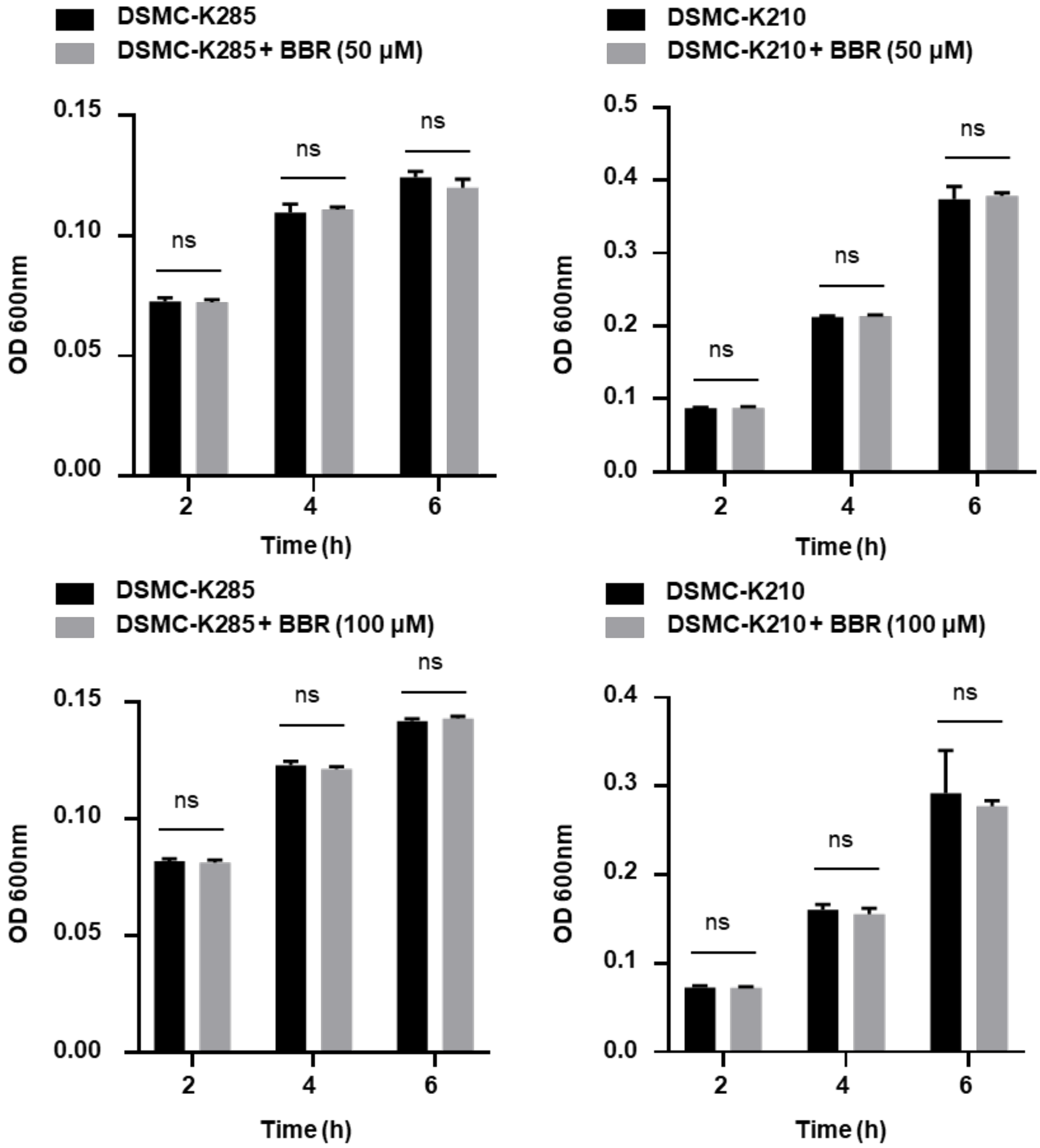

Fig. S1. Berberine treatment shows no effect on hvKp growth. HvKp (DSMC-K285 or DSMC-K210, MOI 40) were incubated with berberine (50 or 100 μM) at various time points in culture medium. All data expressed as the Mean ± SD from three independent experiments (n = 3). Statistical significance was determined using Student's t-test. $^{*}P < 0.05$, $^{**}P < 0.005$, and $^{***}P < 0.001$ according to analysis of variance. ns, not significant; BBR, berberine.
