# Supplementary material for: Berberine promotes host lipolysis to enhance antimicrobial defense against hypervirulent Klebsiella pneumoniae infection
Source: J Microbiol. 2026 Aug 31;64(8):e2605001. doi: 10.71150/jm.2605001 (PMC13688129; doi:10.71150/jm.2605001)
Supplement: Supplementary file 2 [file jm-2605001-Supplementary-Table-S1.pdf]

Table S1. Characteristics of Kp strains using in this work

	**Source**	**Origin**	**String test**	**Antibiotic resistance**	**Serotype**	***rmpA***	***magA***	***allS***	***iutA***	***mrkD***	***entB***	***kfu***
DSMC-K285	Blood	Liver abscess	(+)	Multi-susceptible	K1	(+)	(+)	(+)	(+)	(+)	(+)	(+)
DSMC-K210	Pus	Liver abscess	(+)	Multi-susceptible	K1	(+)	(+)	(+)	(+)	(+)	(+)	(+)
DSMC-K305	Sputum	Pneumonia	(-)	ESBL producer	ND	(-)	(-)	(-)	(-)	(+)	(+)	(-)
