# Supplementary material for: Berberine promotes host lipolysis to enhance antimicrobial defense against hypervirulent Klebsiella pneumoniae infection
Source: J Microbiol. 2026 Aug 31;64(8):e2605001. doi: 10.71150/jm.2605001 (PMC13688129; doi:10.71150/jm.2605001)
Supplement: Supplementary file 3 [file jm-2605001-Supplementary-Table-S2.pdf]

Table S2. Primers used in this study

Genes	Primer	Sequences
Il6	Forward	5′-ACAAAGCCAGAGTCCTTCAGA-3′
	Reverse	5′-TGGTCCTTAGCCACTCCTTC-3′
Tnf	Forward	5′-CCCACGTCGTAGCAAACCAC-3′
	Reverse	5′-GCAGCCTTGTCCCTTGAAGA-3′
Il18	Forward	5′-GACAGCCTGTGTTCGAGGATATG-3′
	Reverse	5′-TGTTCTTACAGGAGAGGGTAGAC-3′
Il1b	Forward	5′-TACGGACCCCAAAAGATGA-3′
	Reverse	5′-TGCTGCTGCGAGATTTGAAG-3′
Map1lc3b	Forward	5′-GTCCTGGACAAGACCAAGTTCC-3′
	Reverse	5′-CCATTCACCAGGAGGAAGAAGG-3′
Sqstm1	Forward	5′-GCAGTTTCCCGACTCCATCTGT-3′
	Reverse	5′-GCTCTTCGGAAGTCAGCAAACC-3′
Gabarap	Forward	5′-AAGAGGAGCATCCGTTCGAGA-3′
	Reverse	5′-GCTTTGGGGGCTTTTTCCAC-3′
Tfeb	Forward	5′-GACTCAGAAGCGAGAGCTAACA-3′
	Reverse	5′-TGTGATTGTCTTTCTTCTGCCG-3′
Acaca	Forward	5′-GATATCCCAGAGATGTTTCGGC-3′
	Reverse	5′-GTCAGCATGTCAGAAGGCAGAG-3′
Scd1	Forward	5′-CCGAAGTCCACGCTCGAT-3′
	Reverse	5′-TGGAGATCTCTTGGAGCATGTG-3′
Fasn	Forward	5′-CACAGTGCTCAAAGGACATGCC-3′
	Reverse	5′-CACCAGGTGTAGTGCCTTCCTC-3′
Pnpla2	Forward	5′-GGAACCAAAGGACCTGATGACC-3′
	Reverse	5′-ACATCAGGCAGCCACTCCAACA-3′
Lipe	Forward	5′-GCTCATCTCCTATGACCTACGG-3′
	Reverse	5′-TCCGTGGATGTGAACAACCAGG-3′
Plin2	Forward	5′-GACAGGATGGAGGAAAGACTGC-3′
	Reverse	5′-GGTAGTCGTCACCACATCCTTC-3′
β-Actin	Forward	5′-CCACCATGTACCCAGGCATT-3′
	Reverse	5′-AGGGTGTAAAACGCAGCTCA-3′
Gapdh	Forward	5′-AAGATGGTGATGGGCTTCCCG-3′
	Reverse	5′-TGGCAAAGTGGAGATTGTTGCC-3′
