# Supplementary material for: Berberine promotes host lipolysis to enhance antimicrobial defense against hypervirulent Klebsiella pneumoniae infection
Source: J Microbiol. 2026 Aug 31;64(8):e2605001. doi: 10.71150/jm.2605001 (PMC13688129; doi:10.71150/jm.2605001)
Supplement: Supplementary file 4 [file jm-2605001-Supplementary-Table-S3.pdf]

Table S3. The information on antibodies

Name	Item number	Dilution rate	Molecular weight	Company
p-p44/42 MAPK (Thr202/Tyr204)	9101	1:1,000	42, 44 kDa	Cell Signaling Technology, USA
p-SAPK/JNK (Thr183/Tyr185)	9251	1:1,000	46, 54 kDa	Cell Signaling Technology, USA
p-mTOR (ser2448)	2971	1:3,000	289 kDa	Cell Signaling Technology, USA
p-p70S6K (Thr421/Ser424)	9204	1:3,000	70, 85 kDa	Cell Signaling Technology, USA
p-S6 (Ser235/236)	2211	1:2,000	32 kDa	Cell Signaling Technology, USA
Perilipin 1	NBP3-16480	1:1,000	56 kDa	Novus Biologicals, USA
TFEB	A303-673A	1:3,000	60 kDa	Bethyl Laboratories, USA
LC3	PD014	1:1,000	16, 18 kDa	MBL, Japan
p62	P0067-200UL	1:3,000	62 kDa	Sigma-Aldrich, USA
p-HSL (Ser563)	4139	1:1,000	81, 83 kDa	Cell Signaling Technology, USA
p-HSL (Ser660)	45804	1:1,000	81, 83 kDa	Cell Signaling Technology, USA
ATGL	2138	1:1,000	54 kDa	Cell Signaling Technology, USA
ACTB	3700	1:5,000	45 kDa	Cell Signaling Technology, USA
Lamin B1	NA12-100	1:2,000	68 kDa	Oncogene, USA
